# Neutralization and Anti-S Antibody Titers in SARS-CoV-2 Boosted Adults in Mexico: A Comparison Across Five Vaccine Types

**DOI:** 10.1155/ijm/6687181

**Published:** 2025-10-10

**Authors:** Nabetse Blas, Martha Carnalla, Ana Basto-Abreu, Jesús Martínez-Barnetche, Celia Alpuche-Aranda, Carlos Gaspar-Castillo, Rodrigo Aparicio-Antonio, Jisela Dimas-González, Irma López, Nayeli Estefanía Sánchez-Casiano, Tonatiuh Barrientos-Gutiérrez

**Affiliations:** ^1^Department of Physical Activity and Healthy Lifestyles, Center for Research in Nutrition and Health, National Institute of Public Health, Cuernavaca, Morelos, Mexico; ^2^Department of Public Health, Faculty of Medicine, National Autonomous University of Mexico, Mexico City, Mexico; ^3^Department of Chronic Diseases, Center for Population Health Research, National Institute of Public Health, Cuernavaca, Morelos, Mexico; ^4^Department of Women's Health, Center for Population Health Research, National Institute of Public Health, Cuernavaca, Morelos, Mexico; ^5^Department of Chronic Infections and Cancer, Center for Research on Infectious Diseases, National Institute of Public Health, Cuernavaca, Morelos, Mexico; ^6^Center for Research on Infectious Diseases, National Institute of Public Health, Cuernavaca, Morelos, Mexico; ^7^Department of Virology, Institute for Epidemiological Diagnosis and Reference, Mexico City, Mexico; ^8^Department of Research, National Center of Blood Transfusion, Mexico City, Mexico; ^9^Diagnostic and Referral, Institute for Epidemiological Diagnosis and Reference, Mexico City, Mexico; ^10^Department of Molecular Microbiology, Institute of Biotechnology, National Autonomous University of Mexico, Cuernavaca, Morelos, Mexico; ^11^Center for Population Health Research, National Institute of Public Health, Cuernavaca, Morelos, Mexico

**Keywords:** antibodies response, COVID-19, heterologous and homologous booster, immunity, Latino population, vaccines

## Abstract

**Introduction:**

The World Health Organization (WHO) recommends the administration of booster doses after completing primary vaccination. Yet, recommendations are largely based on a small set of vaccines, with little information about vaccine mixes used in low and middle-income countries. We aimed to estimate the titers of anti-protein S (RBD) IgG antibodies and neutralization in Mexican adults with a complete vaccination scheme without a booster or with one of five different booster types (heterologous or homologous) to assess immunogenic response.

**Materials and Methods:**

We included data from 2953 adults aged 18 years and older, representing 49.1 million Mexicans, from the National Health and Nutrition Survey (ENSANUT) 2022. We included five primary schemes: BNT162b2, AZD1222, Ad5-nCoV, Gam-COVID-Vac, and CoronaVac. A booster dose was defined as an additional dose beyond the primary scheme and classified as homologous or heterologous based on three vaccine platforms: mRNA, viral vector, and inactivated virus. For each primary scheme, we estimated marginal means of anti-protein S geometric mean titers (GMTs) with 95% CI in each group (no booster, heterologous booster, or homologous booster) adjusted for age, time since the last dose, and prior natural infection using anti-protein N antibodies seropositivity as a proxy using a multivariable linear regression. Then, we estimated the percentage of neutralizing antibodies for the original SARS-CoV-2 strain.

**Results:**

We observed that the highest anti-protein S GMTs were in the group that received a heterologous booster with BNT162b2, AZD1222, Ad5-nCoV, or CoronaVac; the group without a booster showed the lowest GMTs. All groups in the primary schemes had 90% or more inhibition for the original SARS-CoV-2 strain, except in the AZD1222 and Ad5-nCoV groups without a booster.

**Conclusions:**

Boosters increased GMTs for all people, independently of their primary vaccine scheme. Yet, our findings suggest that a heterologous booster produced higher titers against SARS-CoV-2 protein S. Efforts should be made to reach people who received AZD1222 and Ad5-nCoV as their primary scheme and did not get boosted, as neutralization in those groups was particularly low.

## 1. Introduction

COVID-19 still poses a significant risk for vulnerable populations across the world. The Strategic Advisory Group of Experts on Immunization (SAGE) recommended revaccination (beyond the first booster dose) to high-priority groups, such as frontline health workers, elderly individuals, populations with significant comorbidities or severe obesity, immunocompromised patients, and pregnant women [1, 2]. Booster and revaccination are recommended because antibody titers decay over time following vaccination [3–6]. For example, one study found that 6 months after the initial vaccine dose, the observed vaccine efficacy protection declined from 90% to 80% [7, 8]. The declining level of protection could lead to severe outcomes such as hospitalizations and death [8, 9], underscoring the necessity for periodic revaccination.

The evidence supports that applying boosters further improves protection against COVID-19 [10]. Clinical trials have shown that heterologous boosters (i.e., different platform vaccines than the primary scheme) are more immunogenic on average than homologous boosters [11]. For example, a study in Brazil revealed that antibody titers after the administration of a booster dose increased > 75% in the heterologous booster group compared with the homologous group. In China [12], a study reported that heterologous vaccination elicited 5.9-fold (geometric mean titers [GMTs], 197.4 vs. 33.6) higher levels of neutralizing antibodies (Nabs) against the wild-type SARS-CoV-2 and 6.8-fold (GMTs, 53.8 vs. 7.9) for other variants of COVID-19 compared to homologous vaccination. Similar results have been found in Thailand [13], Hong Kong [14], and Turkey [15]. However, most studies have focused on BNT162b2, AZD1222, and CoronaVac vaccines, with few studies analyzing antibody levels in observational settings with a more varied set of combinations of initial vaccines and boosters.

In Mexico, the first COVID-19 vaccines were administered in December 2020, and by 2022, > 90% of the population was vaccinated [1]. The vaccination campaign in Mexico relied on seven different vaccines, depending on availability: BNT162b2 (Pfizer/BioNTech), AZD1222 (AstraZeneca), Gam-COVID-Vac (Sputnik), Ad5-nCoV (CanSino), Antigen SARS-CoV-2 CoronaVac (Sinovac), Janssen/Ad26.COV2.S (Johnson & Johnson), and CX-024414 (Moderna) [16]. This led to different vaccine platform combinations being distributed across the country, leading to a wide range of vaccine combinations for initial vaccination and boosters.

Monitoring the epidemiological situation of vaccines at the population level can help inform which vaccine types may require additional booster doses or the optimal type of booster to enhance protection. We aimed to estimate the titers of anti-RBD IgG and the percentage of Nabs against SARS-CoV-2 in a nationally representative sample of Mexican adults, vaccinated with different combinations of primary and booster vaccine schemes.

## 2. Materials and Methods

### 2.1. Design and Study Population

We used data from the National Health and Nutrition Survey (ENSANUT) conducted from July to December 2022. ENSANUT is a nationally representative population-based survey of Mexico with a probabilistic, multistage, stratified, and clustered sampling design to represent the national, regional (Pacific North, Border, Pacific Center, Center North, Center, Pacific South, Peninsula, Mexico City, and the State of Mexico), and rural/urban levels. Details of ENSANUT's sampling and methodology have been published elsewhere [17]. All questionnaires, interview procedures, and consent forms for ENSANUT were authorized by the Ethics, Research, and Biosafety Committees of the National Health of Public Health (CI: 1807/S7–2022).

**Figure 1:**
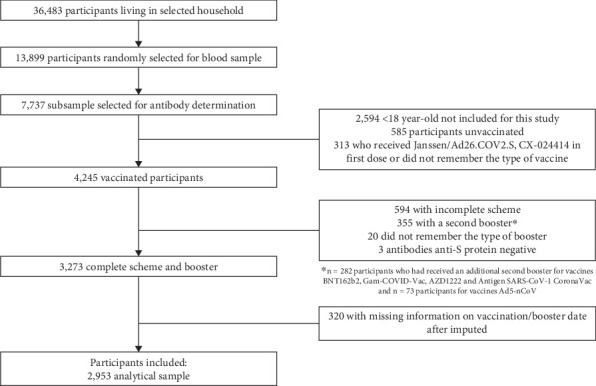
Flowchart of selection process.

The selection process for the participants included in this analysis is detailed in Figure 1. Of the 36,483 selected participants living in a household, a random subsample of 13,899 was selected for blood sampling, of which 7737 participants provided a serum sample for antibody determination. We excluded 2594 participants who were under 18 years old, who reported being unvaccinated, or who did not remember the type of vaccine they received. We excluded participants who received the Janssen/Ad26.COV2.S or CX-024414 as a primary scheme, resulting in 4245 vaccinated adults. We excluded those without a complete primary scheme (*n* = 594) and those who received a second booster (fourth dose for BNT162b2, Gam-COVID-Vac, AZD1222, and Antigen SARS-CoV-2 CoronaVac primary scheme and a third dose for Ad5-nCoV scheme). We further excluded participants who were unsure about their booster vaccine or tested negative for anti-spike protein (S protein) antibodies. From 3273 adults with complete scheme and booster information, we excluded participants who had missing information on vaccination/booster date after imputation (see the Statistical Analysis section). The analytical sample included 2953 adults, representing 49.1 million Mexican adults.

### 2.2. COVID-19 Vaccination

COVID-19 vaccination status was self-reported through the following questions: “Have you received the vaccine for COVID-19?” “How many doses?” and “What type of vaccine did you receive for each dose?” The information was cross-validated with the official certificate, when available. Data for 320 participants could not be validated or imputed and was therefore excluded from the analysis.

#### 2.2.1. Complete Scheme and Booster

We classified individuals as having a complete scheme without a booster and with a homologous or heterologous booster. A complete scheme was defined according to international recommendations at the time of vaccination [18] as follows: two doses for BNT162b2 (Pfizer/BioNTech), AZD1222 (AstraZeneca), Antigen SARS-CoV-2 CoronaVac (Sinovac), and Gam-COVID-Vac (Sputnik) and one dose for Ad5-nCoV (CanSino). A booster dose was defined as an additional dose to the primary scheme of any type of vaccine (i.e., a third dose for two-dose schemes and a second dose for the one-dose scheme) and further classified as homologous or heterologous according to the vaccine platform (mRNA [*BNT162b2 Spikevax*], viral vector [G*am-COVID-Vac*, *AZD1222*, *Ad5-nCoV*, and *Janssen/Ad26.COV2.S*], or inactivated virus [*Antigen SARS-CoV-2 CoronaVac*]) Table 1. A booster was defined as homologous if it was the same vaccine platform as the primary scheme or heterologous if it was different.

### 2.3. Determination of Anti-SARS-CoV-2 Antibodies

We obtained a dried blood spot sample from capillary blood and processed it as previously described [19]. Antibodies directed against the Nucleocapsid (N) and Spike (S) proteins were determined using Roche Elecsys Anti-SARS-CoV-2 pan-immunoglobulin electrochemiluminescence immunoassay (ECLIA) (Roche, Switzerland). The positivity cut-off points for anti-N and anti-S antibodies in DBS were calibrated using a 1752 serum-paired subsample of participants aged 18 years and older. The optimal cut-off after calibration was 0.72 relative units for anti-N and 0.4 for anti-S. Anti-S antibody titers were estimated using the WHO international standard. Further details on this procedure can be found in Section 2.3.1, titled “Serum and Filter Paper Values Correlation.”

#### 2.3.1. Serum and Filter Paper Value Correlation

The National Institute of Public Health (INSP) in collaboration with the Institute of Epidemiological Diagnosis and Reference “Dr. Manuel Martínez Báez” (InDRE), through the application of the ENSANUT in the country, estimated the seroprevalence of COVID-19 in Mexico for the year 2022 by determining the value of IgG antibodies directed against the nucleocapsid protein (N protein) and IgG antibodies directed against the RBD domain of the SARS-CoV-2 S protein from serum samples. COVID-19 vaccines are mostly directed against the SARS-CoV-2 S protein. One of the objectives of measuring these antibodies may be to assess vaccination coverage. However, it is also possible to assess the seroprevalence of natural SARS-CoV-2 infection by determining antibodies against the N protein.

As part of the ENSANUT 2022 activities, 2084 blood samples were initially collected in tubes with vacutainer separator gel and filter paper. The samples were sent to the National Institute of Public Health (INSP) to obtain 6 mm diameter filter paper discs using *PerkinElmer's DBS puncher* equipment. Subsequently, the biological material was sent to the InDRE to validate the feasibility of using filter paper as an alternative method for the collection of biological blood samples. The samples were deep-frozen before analysis. They were analyzed using the ECLIA methodology, and the value of IgG antibodies against SARS-CoV-2 for the N and S proteins was determined.

#### 2.3.2. Sample Processing

To determine the value of antibodies against the N protein and S protein of SARS-CoV-2, paired serum and dried blood spot samples were analyzed. For serum, 200 *μ*L was processed directly in the COBAS E411 equipment following the manufacturer's instructions. Antibody values were expressed as cut-off index (COI) units for anti-N and as units per milliliter for anti-S. For DBS samples in filter paper, two paper discs per sample were used and eluted in 500 *μ*L of buffer (*PBS* + *Tween* 20 + sodium azide), and they were kept at rest for a period of 24 h at a refrigeration temperature (2°C–8°C) until processing using 220 *μ*L of the eluate for determination.

#### 2.3.3. Correction Factor

To extract the equivalent of available serum, the amount of whole blood impregnated in each filter paper disc (DBS) was determined. *PerkinElmer* 226 *Spot Saver Card* filter paper was used for blood sampling during the survey. The saturation point was estimated by impregnating different blood volumes (10–25 *μ*L) on filter paper discs; a double-blind survey determined that the 17 *μ*L disc was at an ideal saturation point. Based on this value, together with the one calculated from an average hematocrit of 43% and 57% serum, 9.69 *μ*L of serum per disc was estimated. With the values of hematocrit and elution volume, the correction factor of 25.8 was obtained, with which each result was adjusted to quantify the real value of antibodies against SARS-CoV-2.

#### 2.3.4. Cohort Points

The positive cut-off point for IgG antibodies against N protein, at which the correction factor is applied, was 0.72 COI; that is, all samples with results less than 0.72 were considered negative. The positive cut-off point for IgG antibodies against protein S was the one established by the manufacturer's insert, that is, 1 mL/U, because the minimum value reported by the team is 0.4 U/mL.

#### 2.3.5. Statistical Analysis

From 1752 paired samples, the degree of agreement between the SARS-CoV-2 antibody results obtained from serum and the results obtained from filter paper was calculated using the kappa concordance test by means of the function *kappa* {base}. To obtain the values of sensitivity, specificity, and positive and negative predictive values (PPV and NPV), the function *BDtest* {bdpv}. For the creation of the ROC curves and the calculation of the area under the curve, the function *Roc* {pROC}. The chi-square test (*χ*^2^) was performed to observe whether or not there is an association between the results obtained by the serum and the filter paper with the function *chisq.test* {stats}.

#### 2.3.6. Results for Consultation

Since the test available for the determination of antibodies against the N protein is qualitative, the value of 0.890 (CI 0.839–0.940) of the kappa test presented the best correlation between the results of the serum samples and filter paper. The SARS-CoV-2 antibody assay for S protein is a quantitative measurement that reports results in units/milliliter. The positivity range is from 0.8 to 250 U/mL, so samples with values higher than the latter were diluted and then adjusted by the dilution factor, which in turn were adjusted by the correction factor. The results obtained by the kappa test indicate an almost perfect degree of agreement between the antibody values against the SARS-CoV-2 N protein in the serum samples and the antibody values against the SARS-CoV-2 N protein in the blood samples preserved on filter paper: 0.890 (CI 0.839–0.940). SARS-CoV-2 S-protein antibody results from serum samples and blood samples preserved on filter paper showed a median degree of agreement of 0.233 (CI −0.032 to 0.499). However, according to the results obtained, there is a statistically significant association between the results against the S protein (23.74, *p* = 1.10e − 06) but not for the N protein (0.006, *p* = 0.9375) (Table 2).

Sensitivity and specificity values were estimated for filter paper samples; for the IgG antibody test against N protein, a sensitivity of 99.14% and a specificity of 92.85% were obtained; for the detection of IgG antibodies against S protein, the sensitivity was 99.12% and a specificity of 100%. However, the optimal specificity of this diagnostic test sacrifices the detection of samples with weak positive values, as observed in 12 DBS samples (Table 3).

**Figure 2:**
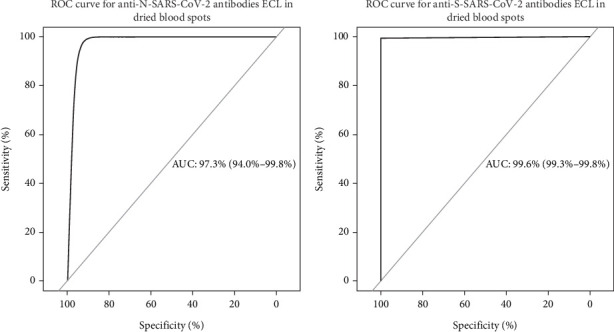
ROC curves for anti-SARS-CoV-2 antibody tests against N and S proteins.

The IgG test against S protein showed better performance in the area under the curve with a result of 99.6% (95% CI 99.3–99.8) compared to the IgG test against protein N, which obtained 97.3% (95% CI 93.9–99.7) of area under the curve. Despite the variation between the two tests, the sensitivity and specificity values are greater than 92.85% (Figure 2).

### 2.4. SARS-CoV-2 Surrogate Virus Neutralization Test

A randomized subsample of serum samples (*n* = 966) was used to determine Nabs against the Wuhan strain of SARS-CoV-2. We used the SARS-CoV-2 Neutralization Antibody Detection Kit (GenScript, Cat. No. L00847-A) according to the manufacturer's instructions [20]. Briefly, sera were diluted at 1:10. RBD antigen coupled with horseradish peroxidase (HRP-RBD) was incubated with diluted sera for 30 min at room temperature. The mixture was added to the wells precoated with ACE2 protein in the 96-well plates for 15 min. After washing, the TMB solution was added to each well. A stop solution was added and read at 450 nm. The inhibition ratio was defined using the average optical density (OD) of the serum sample and the negative control [20].

### 2.5. Covariates

The covariates included at the individual level were sex at birth (male or female), age group in decades (18–29, 30–39, 40–49, 50–59, or 60 or more years old), education (elementary school or less, middle school, high school, or graduate), healthcare institution (covered by social security or uninsured), and employment status (unemployed, student, retired, formal worker, or informal worker). The socioeconomic status classification was based on a principal component analysis of household characteristics and assets to create an index, classified by tertiles into low, medium, or high. At the household level, we included urbanization, which was defined as rural (< 2500 inhabitants), urban (≥ 2500 inhabitants), or metropolitan (≥ 100,000 inhabitants), and region (Pacific North, North Border, Pacific Center, Center North, Center, Pacific South, Peninsula, Mexico City, and the State of Mexico). The months after the last dose were estimated using the reported date of the last dose and the date of the sample collection, but we had 520 (15.8%) missing values. In Mexico, vaccination rollout was by municipality and age in 10-year intervals. That is, the same vaccine type was offered to an age group at a given municipality during a specific time frame. Hence, we imputed the missing values by pairing adults with and without vaccination dates by age group, state of residence, municipality of residence, type of vaccine of the primary scheme, and type of booster (homologous or heterologous). Afterward, 320 (9.7%) remained with missing values and were excluded from the analysis. The sociodemographic characteristics of participants without and with vaccination dates are available in Table 4.

### 2.6. Statistical Analysis

Sociodemographic characteristics were described in percentages (%) and 95% confidence intervals (95% CI). To analyze antibody titers, we estimated the GMTs adjusted by age, months after the application of the last dose, and serostatus of SARS-CoV-2 anti-N IgG as a proxy of previous natural infection. We fitted a multivariable linear model with anti-protein S antibody titers as the dependent variable, type of booster (heterologous, homologous, or no booster) as the independent variable, months since the last vaccine dose, anti-protein N antibodies seropositivity, and age as covariates. Coefficients of variation were used to measure the precision of the estimators. A coefficient of variation of > 30% indicated low precision, and we added a warning in tables or graphs [21]. All analyses were performed using survey weights to consider the sample design using the *svy* module in Stata Version 17 (Stata Corporation, College Station, Texas).

## 3. Results

Our final analytical sample was 2953 participants, which represents 49.1 million Mexican adults.  Table 5 shows the sociodemographic characteristics of Mexican adults with a primary complete vaccination scheme. Women represented 55.7%; the highest proportion was young adults from the age group 18–29 (24.2%) and 30–39 (23.5%), and the lowest proportion was adults from the age group 50–59 (15.9%). Most of the participants had middle (28.1%) or elementary school (26.3%) education. A large proportion of participants lived in the Mexico City/State of Mexico region (24.5%) and the lowest in the Pacific North (8.4%), Center (8.4%), Peninsula (9.0%), and Pacific Center (9.8%). Most of the participants lived in metropolitan areas (51.8%), had a high and middle SES (39.0% and 33.9%), and were informal and formal workers (33.4% and 31.2%). Half of the participants were not affiliated with a social security system (51.4%).

Adults with a heterologous booster had the highest anti-protein S GMTs (Table 6). In BNT162b2 as the primary scheme, participants with a heterologous booster had the highest GMTs (2512.1 IU/mL, 95% CI: 1957.6, 3223.8 IU/mL) in contrast with the other types of boosters. Participants with AZD1222 as the primary scheme and a heterologous booster had higher GMTs with 1616.9 IU/mL (95% CI: 973.4, 2686.0 IU/mL) than participants with a homologous booster or with no booster (830.3 IU/mL [95% CI: 662.6; 1040.5 IU/mL] or 718.5 IU/mL [95% CI: 573.9; 899.6 IU/mL]). Higher GMTs were also observed in those who received a heterologous booster and had Antigen SARS-CoV-2 CoronaVac or Ad5-nCoV as the primary scheme (1509.3 IU/mL [95% CI: 753.2; 3024.5 IU/mL] and 1495.6 IU/mL [95% CI: 736.5; 3036.8 IU/mL]), but the coefficient of variation for Antigen SARS-CoV-2 CoronaVac or Ad5-nCoV was high (39% and 38%). Participants with Ad5-nCoV as the primary scheme who had received a homologous booster or no booster showed the lowest GMTs across all combinations (839.1 IU/mL [95% CI: 471.7; 1492.7 IU/mL] and 656.0 IU/mL [95% CI: 325.1; 1323.8 IU/mL], respectively). The lowest GMTs were observed in those with no booster and who had Gam-COVID-Vac or Antigen SARS-CoV-2 CoronaVac as the primary scheme (713.1 IU/mL [95% CI: 344.8; 1474.9 IU/mL] and 575.3 IU/mL [95% CI: 272.0; 1217.1 IU/mL]).

AZD1222 and Ad5-nCoV groups showed Nabs of 84.3% (95% CI: 74.5, 94.2) and 88.7% (95% CI: 78.4, 99.1), respectively, among participants who did not receive a booster dose, whereas those who received a booster or other vaccines showed Nabs of at least 90% (Table 6). The distribution of the type of vaccines in the primary and booster regimen is available in Table 6.

## 4. Discussion

We aimed to estimate anti-protein S IgG titers and the percentage of Nabs in serums from a nationally representative sample of Mexican adults with a complete vaccination scheme and a heterologous, homologous, or no booster dose. We found that in adults aged 18 and older, the highest anti-protein S GMTs were in the group that received a heterologous booster with a primary scheme with BNT162b2, AZD1222, Ad5-nCoV, and Antigen SARS-CoV-2 CoronaVac. In all the vaccine schemes, the group with no booster showed the lowest GMTs. Our findings suggest that a heterologous booster in participants with a complete scheme had higher titers against SARS-CoV-2 protein S. Overall, all the groups showed a high percentage of viral neutralization. However, it is important to note that participants who did not receive a booster of the AZD1222 and Ad5-nCoV vaccines had lower percentages than the rest < 90%.

Since the early days of the implementation of SARS-CoV-2 vaccines, doubts have arisen regarding vaccine efficacy and the need for boosters [22]. In our analysis, the greatest difference in anti-protein S GMTs was observed between participants having a booster dose and not having one. Our findings support the data indicating that boosters generate higher antibody levels and may also help prolong protection over time, reduce the transmission of the virus, and contribute to the prevention of new cases or less severe infections [23]. Our analysis provides much-needed information about vaccines that have not been frequently studied, such as Ad5-nCoV and Gam-COVID-Vac, but that have been used in Mexico. Those specific vaccines also benefited similarly from boosters. Furthermore, beyond the results observed in enhanced immune response due to boosters in our study, it is crucial to consider how these findings may inform public health policies. Evidence suggests that boosters not only increase antibody levels but may also play a crucial role in reducing virus transmission and preventing new cases [10]. However, addressing equity in booster access, particularly in resource-limited regions, is essential to ensure equitable distribution and maximize the benefit across all population groups. However, it is important to highlight the need and optimal time to administer boosters, as they may depend on several factors that determine the application of the vaccine, including the right to vaccination and vaccine availability.

Evidence available for WHO-approved vaccines suggests that heterologous boosters may induce higher antibody titers than a homologous booster or no booster and could promote a better immune response against SARS-CoV-2 [24, 25]. Most of the evidence comes from randomized clinical trials. In Spain [26], a multicenter, randomized, active-controlled, double-blind, noninferiority Phase IIb trial involving adult participants showed a higher GMTs ratio in heterologous booster 1.68 (*p* < 0.0001), 1.31 (*p* = 0.0007), and 0.86 (*p* = 0.40) on Days 14, 28, and 98, respectively, for the first strain of SARS-CoV-2. In Japan [27], a Phase III, double-blind, multicenter, randomized, controlled, noninferiority trial conducted at 11 outpatient clinical centers found that healthy adults who received a heterologous booster dose reported higher GMTs with 5641 IU/mL (95% CI: 4321, 7363 IU/mL) when measured at 28 days after the booster. In India [28], a randomized Phase III study observed a stable increase in GMTs with 26717.7 (95% CI: 24,628.78, 28,983.83) in a population that received timely heterologous boosters. A further study in Indonesia [29] retrospectively evaluated the antibody response in healthcare workers who received a booster dose of mRNA and inactivated vaccines and reported that the total antibody titers (43,472 U/mL) after receiving a heterologous booster were significantly higher than when they did not receive a booster. In Latin America, a Phase IV clinical trial in Brazil found that heterologous booster vaccination improved immune responses by increasing anti-protein S antibodies titers and could better protect against SARS-CoV-2 infection [11]. The observed increase in GMTs shows that there is a greater probability of protection against SARS-CoV-2 and possible strains if the population receives a heterologous booster. Research is still ongoing, and evidence from population studies is limited. Our results concur with these studies but provide evidence from a national survey of the Mexican population in a real vaccination scenario with different types of vaccines used in middle-income countries.

This enhanced immunity is attributed to the use of distinct vaccine platforms that stimulate complementary arms of the immune system [30]. For example, AZD1222 has been shown to induce strong cellular immunity [31], while BNT162b2 elicits potent humoral responses [32]. The sequential activation of different innate and acquired immunity arms leads to broader and more robust activation of B and T lymphocytes, resulting in higher titers of Nabs and stronger cellular responses. Due to antigen-specific T cells being more conserved among SARS-CoV-2 strains than antigens recognized by antibodies [33–37], cellular immunity has been associated with cross-variant immunity, contributing to reduced severity and mortality for new strains [38–40].

The preference for a heterologous booster over a homologous one is important to consider. Incorporating COVID-19 heterologous booster vaccines may improve protection against infection and influence the breadth of vaccine-elicited antibodies by including different antigens, vectors, doses, or adjuvants at different times [12]. Most evidence available for WHO-approved vaccines indicates that a heterologous booster is safe, has better immune responses, and is more effective than a homologous booster, and it also highlights the potential benefits for public health strategies [23] that are particularly relevant for low and middle-income countries. By considering heterologous boosters, healthcare systems, key stakeholders, and policymakers gain flexibility for vaccination campaigns, particularly when faced with challenges such as supply chain constraints [22, 41, 42]. Additionally, the choice of a heterologous booster could prove advantageous for individuals with varying vaccination histories, potentially offering a robust and adaptable solution over time [43]. Further studies and comprehensive evaluation of this approach will be essential to elucidate the full spectrum of benefits and to optimize booster vaccination strategies for a wider population.

We observed significant variations in the distribution of sociodemographic characteristics, such as age, educational level, and geographic region. While the primary analysis focused on the efficacy of the heterologous versus homologous booster regimen, it is important to acknowledge that these covariates also play a critical role in vaccination dynamics and immune response. For instance, the higher proportion of participants in metropolitan areas and with higher socioeconomic status may influence access to and acceptance of vaccination regimens, while differences in age and educational level may be associated with variations in immune response. These disparities highlight the need for a tailored approach to vaccination strategies that address regional and sociodemographic inequalities to improve vaccination coverage and efficacy among the Mexican population.

Our study has several limitations. First, the information on vaccination (type and date) was self-reported by a key household informant, which could introduce error; however, the information was validated with the official certificate when available. Furthermore, we do not expect to have a differential recall conditional on antibody titers or vaccine type. Second, the results are reported one time in a cross-sectional study following a short time. However, our results are at the population level from a national survey (ENSANUT). Third, for some vaccine types, sample sizes were small, and the estimators had high uncertainty; hence, those results should be interpreted with caution. Fourth, we acknowledge that prior COVID-19 infection could influence antibody titers. In our study, 603 participants reported having contracted COVID-19 between February 2020 and December 2022. We have adjusted our analyses for prior infection using anti-protein N seropositivity as a proxy of infection. Our study analyzed neutralization through the percentage of inhibition using the Wuhan strain of SARS-CoV-2; prior studies evaluating heterologous vaccination regimens have shown that neutralization against the Wuhan strain is not equivalent to neutralization against Omicron and its sublineages [44, 45]. However, in the current context, evaluating neutralization against the Omicron strain in population-based studies is challenged due to the wide variety of vaccine platforms used in Mexico and the multiple waves of SARS-CoV-2 infection that the population has experienced. Finally, although we measured antibody titers and neutralization capacity, these immunological markers do not directly indicate clinical protection against infection or severe disease. However, our findings provide an alternative perspective in the event of a future outbreak, as higher immune protection could potentially reduce severe illness and help mitigate the spread of infection. Therefore, our results should be interpreted as indicators of population-level immune response rather than predictors of individual-level clinical outcomes. Further studies should analyze current neutralization using standards for new SARS-CoV-2 variants.

## 5. Conclusions

Receiving a booster dose increased the anti-protein S GMTs regardless of the primary vaccine scheme. Higher GMTs for anti-protein S antibodies were observed in individuals receiving heterologous booster doses compared to those receiving homologous booster or without a booster. This nationally representative study provides evidence for public health research and prevention strategies. Ongoing efforts are needed to encourage people to revaccinate, especially those who received AZD1222 and Ad5-nCoV as the primary scheme and did not get boosted, as neutralization of the original SARS-CoV-2 strain in that group was particularly low.

## Figures and Tables

**Table 1:** Availability of vaccines for SARS-CoV-2 Wuhan strain in Mexico in 2022.

**Type vaccine**	**Biopharmaceutical name**	**Design platform vaccine**	**Primary complete scheme**	**Booster**
Pfizer	BNT162b2	mRNA	2 doses	1 dose
Moderna	CX-024414	mRNA	2 doses	1 dose
Sputnik	Gam-COVID-Vac	Viral vector	2 doses	1 dose
AstraZeneca	AZD1222	Viral vector	2 doses	1 dose
CanSino	Ad5-nCoV	Viral vector	1 dose	1 dose
Johnson & Johnson	Janssen/Ad26.COV2.S	Viral vector	2 doses	1 dose
Sinovac	Antigen SARS-CoV-2 CoronaVac	Inactivated virus	2 doses	1 dose

**Table 2:** Cohen's kappa coefficient.

**Sample**	**X** ^2^	**Mexico City**	**p** **value**	**Kappa value**
Serum N vs. filter N	0.0061	1	0.9375	0.890 (CI 0.839–0.940)
Serum S vs. filter S	23.74	1	1.1*e* − 06	0.233 (CI −0.032 to 0.499)

*Note:* N, protein N; S, protein S.

**Table 3:** Sensitivity, specificity, PPV, NPV, and AUC.

**Immunoassay**	**Sensitivity**	**Specificity**	**VPP**	**VPN**	**AUC**
ECLIA on filter paper N	99.14 (95% CI 98.5–99.5)	92.85 (95% CI 85.0–97.3)	99.57 (95% CI 99.0–99.8)	98.67 (95% CI 78.6–91.9)	97.3 (95% CI 93.9–99.7)
ECLIA on filter paper S	99.12 (95% CI 98.5–99.5)	100 (95% CI 15.8–100)	99.95 (95% CI 99.8–99.9)	9.39 (95% CI 4.2–16.7)	99.6 (95% CI 99.3–99.8)

*Note:* AUC, partial area under the concentration–time curve. A statistical significance of *p* < 0.05 is assumed.

**Table 4:** Sociodemographic characteristics distribution in Mexican adults with and without information on vaccination or booster date, ENSANUT 2022.

	**Without date**		**With date**		
	**n** = 520		**n** = 2754		
	**n**	**%**	**n**	**%**	**p** **value**
Sex					0.11
Men	60	30	977	35.5	
Women	140	70	1777	64.5	
Age group (years)					0.41
18–29	31	15.5	576	20.9	
30–39	40	20	484	17.6	
40–49	37	18.5	530	19.2	
50–59	42	21	540	19.6	
60 and more	50	25	624	22.7	
Education					0.62
Elementary school or less	56	28	889	32.3	
Middle school	66	33	867	31.5	
High school	44	22	586	21.3	
Graduate	34	17	412	15	
Region					< 0.001
Pacific North	27	13.5	255	9.3	
North Border	55	27.5	543	19.7	
Pacific Center	18	9	209	7.6	
Center North	38	19	576	20.9	
Center	26	13	229	8.3	
Mexico City/State of Mexico	19	9.5	324	11.8	
Pacific South	7	3.5	254	9.2	
Peninsula	10	5	364	13.2	
Location area					< 0.001
Rural	34	17	769	27.9	
Urban	47	23.5	730	26.5	
Metropolitan	119	59.5	1255	45.6	
Socioeconomic status (SES)					< 0.001
Low	45	22.5	880	32	
Middle	66	33	953	34.6	
High	89	44.5	921	33.4	
Social security					0.01
No	85	42.5	1454	52.8	
Yes	115	57.5	1300	47.2	
Employment status					0.01
Unemployed^a^	73	36.5	933	33.9	
Student	7	3.5	115	4.2	
Retired	19	9.5	119	4.3	
Informal worker	45	22.5	822	29.9	
Formal worker^b^	56	28	765	27.8	

*Note: n*, sample size; *N*, expanded sample.

^a^Includes all nonpaid workers, houseworkers, and individuals that are unable to work due to disability.

^b^Paid workers with access to formal security healthcare.

**Table 5:** Sociodemographic characteristics of Mexican adults with a primary COVID-19 vaccination scheme, ENSANUT 2022.

**n** = 2953, **N** = 49.1** (million)**	**%**	**(95% CI)**
Sex		
Men	44.3	(41.0; 47.5)
Women	55.7	(52.4; 58.9)
Age group (years)		
18–29	24.2	(21.6; 27.0)
30–39	23.5	(20.6; 26.7)
40–49	17.9	(15.5; 20.6)
50–59	15.9	(14.1; 17.8)
60 and more	18.3	(16.3; 20.4)
Education		
Elementary school or less	26.3	(23.6; 29.0)
Middle school	28.1	(25.6; 30.6)
High school	24.9	(22.5; 27.5)
Graduate	20.7	(18.1; 23.5)
Region		
Pacific North	8.4	(7.0; 10.0)
North Border	14.2	(12.8; 15.7)
Pacific Center	9.8	(8.2; 11.5)
Center North	13.2	(11.9; 14.5)
Center	8.4	(6.7; 10.2)
Mexico City/State of Mexico	24.5	(20.0; 27.1)
Pacific South	12.5	(10.7; 14.5)
Peninsula	9	(7.8; 10.4)
Location area		
Rural	19.7	(17.3; 22.2)
Urban	28.6	(26.4; 30.8)
Metropolitan	51.8	(49.1; 54.4)
Socioeconomic status (SES)		
Low	27.2	(24.5; 29.9)
Middle	33.9	(31.1; 36.5)
High	39	(35.7; 42.4)
Social security		
No	51.4	(48.4; 54.3)
Yes	48.6	(45.6; 51.5)
Employment		
Unemployed^a^	27	(24.9; 29.0)
Student	4.6	(3.5; 5.9)
Retired	3.9	(3.0; 4.9)
Informal worker	33.4	(30.8; 36.0)
Formal worker^b^	31.2	(28.2; 34.2)

*Note: n*, sample size; *N*, expanded sample.

^a^Includes all nonpaid workers, houseworkers, and individuals that are unable to work due to disability.

^b^Paid workers with access to a formal security healthcare.

**Table 6:** Anti-protein S geometric mean antibody titers in Mexican adults with a primary complete scheme and type of booster, ENSANUT 2022.

**Primary complete scheme**	**Booster**	**n**	**GMTs ** ^**h**^ ** (95% CI)**	**p** **value**	**n**	**Inhibition % (95% CI)**
BNT162b2				⁣^∗^		
Pfizer	Homologous^a,b^	261	1689.6 (1243.9; 2294.9)		94	94.3 (91.9; 96.8)
	Heterologous^c,d,e^	378	2512.1 (1957.6; 3223.8)		127	94.9 (93.7; 96.1)
	No booster	339	1876.7 (1482.7; 2375.3)		112	92.9 (90.3; 95.4)
AZD1222				⁣^∗^		
AstraZeneca	Homologous^c,d,e^	767	830.3 (662.6; 1040.5)		234	91.3 (88.1; 94.4)
	Heterologous^a,b,c,d,e^	32	1616.9 (973.4; 2686.0)		16	96.5 (96.1; 96.9)
	No booster	422	718.5 (573.9; 899.6)		138	84.3 (74.5; 94.2)
Ad5-nCoV				⁣^∗^		
CanSino	Homologous^c,d,e,f^	120	839.1 (471.7; 1492.7)⁣^∗∗^		39	90.1 (84.5; 95.6)
	Heterologous^a,b^	46	1495.6 (736.5; 3036.8)⁣^∗∗^		18	93.8 (90.4; 97.1)
	No booster	118	656.0 (325.1; 1323.8)⁣^∗∗^		50	88.7 (78.4; 99.1)
Antigen SARS-CoV-2 CoronaVac				⁣^∗^		
Sinovac	Homologous^g^	32	1246.6 (576.4; 2695.9)⁣^∗∗^			
	Heterologous^a,b,c,d,e^	182	1509.3 (753.2; 3024.5)⁣^∗∗^		65	92.9 (89.0; 96.9)
	No booster	88	575.3 (272.0; 1217.1)⁣^∗∗^		26	91.9 (88.8; 95.1)
Gam-COVID-Vac						
Sputnik	Homologous^c,d,e^	98	1215.8 (617.5; 2393.8)⁣^∗∗^		16	95.3 (93.3; 97.3)
	No booster	52	713.1 (344.8; 1474.9)⁣^∗∗^		21	94.1 (91.4; 96.7)

*Note: n*, sample size.

Abbreviation: GMT, geometric mean titer.

^a^BNT162b2.

^b^CX-024414.

^c^Gam-COVID-Vac.

^d^AZD1222.

^e^Ad5-nCoV.

^f^Janssen/Ad26.COV2.S.

^g^Antigen SARS-CoV-2 CoronaVac.

^h^Adjusted by months since the last dose, anti-N protein antibodies positivity, and age in years.

⁣^∗^*p* value < 0.05. ⁣^∗∗^Coefficient of variation > 30%.

## Data Availability

The code and database of this analysis are available at GitHub: blasnabetse/Booster-and-SARS-CoV-2-in-Mexico-in-2022 (https://github.com/).
